# HOXC10 promotes growth and migration of melanoma by regulating Slug to activate the YAP/TAZ signaling pathway

**DOI:** 10.1007/s12672-021-00408-7

**Published:** 2021-04-26

**Authors:** Yuanxin Miao, Weina Zhang, Su Liu, Xiangfeng Leng, Chunnan Hu, Hao Sun

**Affiliations:** 1 Department of Plastic Surgery, The Affiliated Hospital of Qingdao University, Qingdao, 260003 Shandong China; 2 Department of Environmental Art Design, Qingdao University of Science and Technology, No. 99 Songling Road, Qingdao, 260061 Shandong China

**Keywords:** HOXC10, Slug, YAP/TAZ, Melanoma

## Abstract

Homeobox C10 (HOXC10) has been reported to participate in various cancers. However, the involvement of HOXC10 in melanoma is still unknown. Here, we attempted to determine whether HOXC10 can affect the development of melanoma. We separated melanoma tissues and the matched tumor-adjacent normal tissues from melanoma patients, and examined HOXC10 expression in the melanoma cells and tissues. Comparing with the tumor-adjacent normal tissues, HOXC10 was up-regulated in melanoma tissues. Melanoma cells also displayed an up-regulation of HOXC10. Moreover, HOXC10 inhibition suppressed cell proliferation, clone formation and promoted apoptosis of melanoma cells. Knockdown of HOXC10 also retarded migration, invasion and epithelial–mesenchymal transition (EMT) in melanoma cells. Additionally, HOXC10 accelerated Slug expression by interacting with Slug, and activating the promoter of Slug. Slug activated the YAP/TAZ signaling pathway, which was reversed by HOXC10 silencing. The in vitro assays demonstrated that inhibition of HOXC10 significantly repressed tumor growth and lung metastasis of melanoma in mice by inhibiting Slug and YAP/TAZ signaling pathway. In conclusion, this work demonstrated that HOXC10 promoted growth and migration of melanoma by regulating Slug to activate the YAP/TAZ signaling pathway. Therefore, this study suggests that inhibition of HOXC10 has therapeutic potential in melanoma.

## Introduction

Melanoma is an malignant epithelial tumor that originates from the melanocytes of neural crest, that is, a malignant change in pigmented nevus [1]. Melanoma is a highly malignant, most aggressive and lethal tumor among human skin cancer. It originates from the imbalance of melanocyte proliferation, which accounts for more than 80% of skin cancer-related deaths [2]. The latest research conclusions point out that long-term exposure to strong sunlight, genetic mutations and environmental factors may cause the malignant transformation of nevus and pigmented spots into melanoma [3, 4]. In the past decade, scholars have conducted a large number of researches and clinical trials to explore the potential molecular pathogenesis and treatment strategies of melanoma. At present, it has been proved that various pathways are associated with the occurrence of melanoma, such as MAPK, PI3K/Akt and YAP/TAZ pathways [5–8].

Homeobox C10 (HOXC10), as a member of the homeobox gene family, can significantly enhance cell proliferation in cancer, and can be used as a marker for cancer diagnosis or progression evaluation [9]. Accumulation studies have found that HOXC10 is abnormally expressed in various tumors. For example, HOXC10 is highly expressed in glioblastoma, and HOXC10 upregulation activates PI3K/AKT signaling pathway to promote glioblastoma cell proliferation and accelerate poor prognosis of glioblastoma [10]. In gastric cancer, up-regulation of HOXC10 promotes the proliferation and metastasis of gastric cancer cells by regulating NF-κB or MAPK pathway [11–13]. Overexpressed HOXC10 can interact with PRMT5 to promote the expression of VEGF, thereby promoting angiogenesis in gliomas and increasing the metastasis and invasion abilities of gliomas [14]. HOXC10 can also fine-tune DNA repair to promote drug resistance in breast cancer cells [15]. In addition, TCGA and CCLE database reveal that HOXC10 is highly expressed in the tumor tissues of melanoma. However, the mechanism of action of HOXC10 in the progression of melanoma is still remains unclear, and further research is still needed in melanoma.

Slug is an epithelial–mesenchymal transition (EMT)-related protein, and its upregulation causes EMT to promote tumor migration and invasion [16, 17]. Previous study has shown that HOXC10 interacts with Slug, and increases Slug transcription, thereby causing the migration of ovarian cancer cells [18]. Slug is also participates in the development of melanoma and increases the metastasis of melanoma [19, 20]. Additionally, Slug interacts with YAP to active the YAP/TAZ signaling pathway, thereby controlling the self-renewal and differentiation of skeletal stem cells [21]. Therefore, this work attempted to investigate whether HOXC10 can regulate Slug expression to activate the YAP/TAZ signaling pathway, thereby promoting the growth and metastasis of melanoma.

## Materials and methods

### Participants

We recruited melanoma patients who underwent surgical removal of melanoma at Affiliated Hospital of Qingdao University. Melanoma tissues and the matched tumor-adjacent normal tissues (n = 60) were obtained from the surgery and stored further use. These patients with melanoma did not receive any treatment before surgery. All melanoma patients were informed and gave written the consent. All protocols were performed according to the Declaration of Helsinki and authorized by the Ethics Committee of The Affiliated Hospital of Qingdao University.

### Cell culture

Normal human dermal fibroblast cells (NHDF, ATCC, Manassas, VA, USA) and melanoma cell lines (A2058, A375, WM115, MV3, ATCC) were cultured in Dulbecco's modified Eagle’s medium (DMEM, Gibco, Waltham, MA, USA) at a constant temperature (37 °C) with 5% CO_2_. The medium was contained 10% fetal bovine serum (FBS, Gibco) and 1% penicillin/streptomycin (Solarbio, Beijing, China).

### Cell transfection

The vector pcDNA3.1 containing HOXC10 (pcDNA3.1-HOXC10) or Slug (pcDNA3.1-Slug) were constructed for HOXC10 or Slug overexpression (GenePharma, Shanghai, China). The vector pcDNA3.1-NC was served as negative control (NC). For HOXC10 knockdown, HOXC10 (sh-HOXC10) targeted short hairpin RNA (shRNA) and the scramble shRNA (shNC) were synthesized by GenePharma. Cells were transfected with the vectors or oligonucleotides using Lip2000 (Invitrogen, Carlsbad, CA, USA).

### Gene expression

Total RNA was extracted applying Trizol reagent (Invitrogen). 1.0% agarose gel electrophoresis was used to examine RNA integrity. The relative expression of genes in cells and tissues was examined through quantitative real-time PCR (qRT-PCR) using TransScript® II Two-Step RT-PCR SuperMix (Transgen, Beijing, China) on a Real-Time PCR Instrument. The 2^−ΔΔCt^ method was used to analyze the data. Primer sequences (5′-3′) were as follows:

HOXC10-F: AGAGCACAGGCAGAATCGTT;

HOXC10-R: GCTCTGGGTGCTACGACAAA;

Slug-F: GGGGAGAAGCCTTTTTCTTG;

Slug-R: TCCTCATGTTTGTGCAGGAG;

GAPDH-F: CGGATTTGGTCGTATTGGG;

GAPDH-R: GATTTTGGAGGGATCTCGC.

### Protein expression

Protein extraction and the detection of protein concentration were carried out using Tissue or Cell Total Protein Extraction Kit (Sangon Biotech, Shanghai, China) and BCA Protein Assay Kit (Sangon Biotech). Proteins (25 μg) were used for 10% SDS-PAGE gel electrophoresis, and then blotted onto the nitrocellulose membranes (Whatman, Maidstone, Kent, UK). The membranes were stained with the primary antibodies, HOXC10 (1:1000, #ab153904), Bax (1:2000, #ab182733), BCL2 (1:1000, #ab32124), Cleaved Caspase-3 (1:500, #ab2302), E-Cadherin (1:1000, #ab133597), N-Cadherin (1:5000, #ab76011), snail (1:1000, #ab216347), Slug (1:1000, #ab183760), YAP1 (1:5000, ab52771) and TAZ (1:1000, #ab224239) at 4 °C for 12 h. Subsequently, the membranes were stained with HRP-IgG (1:2000, #ab6721). β-actin antibody (1:1000, # ab5694) was used as an reference protein. The data were analyzed by Image J software. These antibodes all obtained from Abcam (Cambridge, MA, USA).

### Immunohistochemical (IHC) staining

Paraffin tissue sections were prepared through fixation and embedding. After deparaffin and rehydration, the sections were incubated with Target Retrieval Solution (Dako, CA, USA). The sections were stained with the primary antibodies, HOXC10 (1:1000, #ab153904, Abcam), anti-Slug (1:100, #ab85936, Abcam), anti-YAP1 (1:50, #ab52771, Abcam) and anti-TAZ(1:500, #ab224239, Abcam), and then labeled with HRP-IgG (1:2000, #ab6721, Abcam). Finally, the sections were stained successively with DAB and hematoxylin. The sections were observed under a microscope (Olympus, Tokyo, Japan).

### Cell viability

Cell viability was examined using Cell Counting Kit-8 (Beyotime Biotechnology, Shanghai, China). Cells (2000 cells/100 μL) were seeded into 96-well plate, and incubated with 10 μL CCK-8 reagent at 37 °C for 72 h. The absorbance value of each well was detected at 450 nm using a microplate reader (Bio-Rad, Hercules, CA, USA).

### Cell proliferation

Cell proliferation was examined using Yefluor 488 EdU Imaging Kit (Yeasen, Shanghai, China). Cells were first incubated with EdU at 37 °C for 4 h. After fixation and permeabilization, the cells were reacted with 1 mL Click-iT reaction cocktail in darkness for 30 min. For nuclear staining, cells were incubated with DAPI. Finally, the fluorescence was observed under a fluorescence microscope (Nikon, Tokyo, Japan).

### Cell clone formation assay

Cells at logarithmic phase were treated with 0.25% trypsin/0.02% EDTA to obtain the single-cell suspension. The cells (1000/well) were seeded into a six-well plate, and cultured at 37 °C. Until the cell clones could be observed with the naked eye, the cells were stopped. After fixation, the cells were stained with Giemsa solution (Beyotime Biotechnology) for 30 min. Finally, the number of cell clones was counted.

### Apoptosis

Cell apoptosis was examined using Annexin V-FITC Apoptosis Detection Kit (Beyotime Biotechnology). After washing with PBS, cells were mixed with Annexin V-FITC binding buffer, and then stained with Annexin V-FITC and PI at darkness for 20 min. Finally, cell apoptosis was examined on a FACSCalibur (BD Biosciences, San Jose, CA, USA) in an hour.

### Migration and invasion

Cell migration and invasion were estimated using a 24-well Transwell insert system (Corning, NY, USA). For migration assay, cells were cultured in the DMEM-contained upper chamber. For invasion assay, cells were cultured in the upper chamber containing DMEM and coating with Matrigel. The bottom chamber was supplemented with DMEM and 10% FBS. After 24 h of culture, the migrating or invading cells on the bottom surface of the chamber were collected, and then were fixed with paraformaldehyde and dyed with 0.1% crystal violet. Images were photographed from each membrane under Olympus microscope from five randomly selected fields.

### Co-immunoprecipitation

The relationship between HOXC10 and Slug in the HOXC10-overexpressed A375 and A2058 cells was determined by Co-Immunoprecipitation (Co-IP) assay. The washed cells were lysed with RIPA Lysis Buffer (Beyotime, Shanghai, China) at 4 °C for 30 min. Subsequently, the cell lysates were incubated with the primary antibodies, HOXC10 (1:100, #A303-177A, Thermo Fisher Scientific) or Slug (1:50, #9585, Cell Signaling Technology, Danvers, MA, USA), and then incubated with the Protein A/G Plus-Agarose (Thermo Fisher Scientific). Immunoprecipitates and a portion of the cell lysates (Input group) were analyzed by WB assay using the primary antibodies, HOXC10 and Slug.

### Luciferase reporter assay

The interaction between HOXC10 and Slug in A375 and A2058 cells was examined by luciferase reporter assay. The vector pGL3-basic containing slug promoter (pGL3-basic-slug promoter) was transfected into cells using Lip2000 Reagent. Cells were co-transfected with pGL3-basic-slug promoter and different concentration of pcDNA3.1-HOXC10 (0, 50, 100, 200 ng). The activities of luciferase were measured using Dual luciferase assay kit (Promega, Madison, USA) on luciferase assay system (Ambion, Austin, TX, USA).

### Xenograft mouse model

Female BALB/c nude mice (SLAC, Shanghai, China) (weighting 18 ± 2 g, aged 4 weeks) were housed at 18–25 °C. All the animal protocols were carried out according to the Guide for the Care and Use of Laboratory Animals, and authorized by the Ethics Committee of The Affiliated Hospital of Qingdao University. For tumor xenograft mouse model, HOXC10-silenced A375 cells (200 µL, 2.0 × 10^6^/mL) were injected subcutaneously into the nude mice. The mice were euthanized to separate the tumor xenografts, the weight and volume of tumor were measured every week for 28 days. Volume (mm^3^) = 1/2 × width^2^ (mm^2^) × length (mm). For metastasis mouse model, HOXC10-silenced A375 cells (200 µL, 2.0 × 10^6^/mL) were injected into the caudal vein of nude mice. After 28 days, the mice were euthanized to obtain the lung tissues. HE staining was performed to estimate the number of metastatic tumors in the lungs.

### HE staining

Lung tissues of mice were embedded in paraffin to obtain paraffin sections. The paraffin sections (5 μm) were stained using HE staining kit (Solarbio, Beijing, China). The number of metastases in lung tissues was observed under an Olympus microscope.

### Statistical analysis

All assays were carried out for 3 times. Statistical analysis was done through SPSS 20.0 (SPSS, Inc., Chicago, IL, USA) or GraphPad Prism 7.0 software (San Diego, CA, USA, USA). The data were showed as the mean ± SD. Statistical differences were carried out through the Student’s t-test or one-way ANOVA. *p* < 0.05 was supposed as statistically significant.

## Results

### HOXC10 was highly expressed in melanoma tissues and cells

**Fig. 1:**
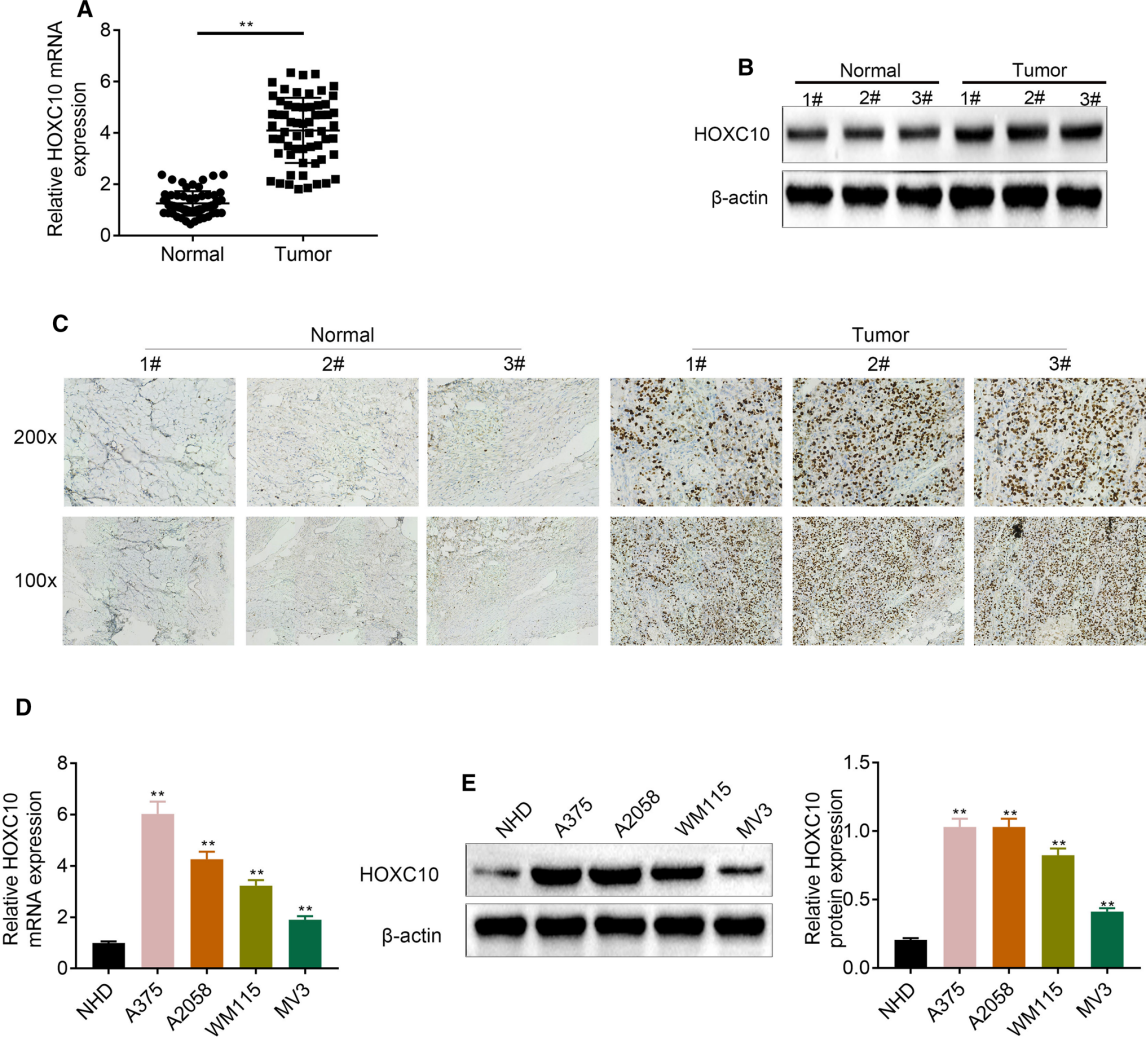
HOXC10 was highly expressed in melanoma tissues and cells. HOXC10 expression in melanoma tissues was assessed through qRT-PCR (**a**), WB (**b**) and IHC staining (**c**). HOXC10 expression in NHDF, A375, A2058, WM115 and MV3 cells was estimated through qRT-PCR (**d**) and WB (**e**). ^**^p < 0.01

We initially compared HOXC10 expression between melanoma tissues and tumor-adjacent normal tissues of patients with melanoma. As shown in Fig. 1a–c, these results all showed that HOXC10 mRNA and protein expression was up-regulated in melanoma tissues of melanoma patients. HOXC10 mRNA and protein expression was also significantly increased in A375, A2058, WM115 and MV3 cells with respect to NHDF cells (Fig. 1d, e). Thus, up-regulation of HOXC10 may be associated with melanoma development.

### HOXC10 promoted proliferation of melanoma cells

**Fig. 2:**
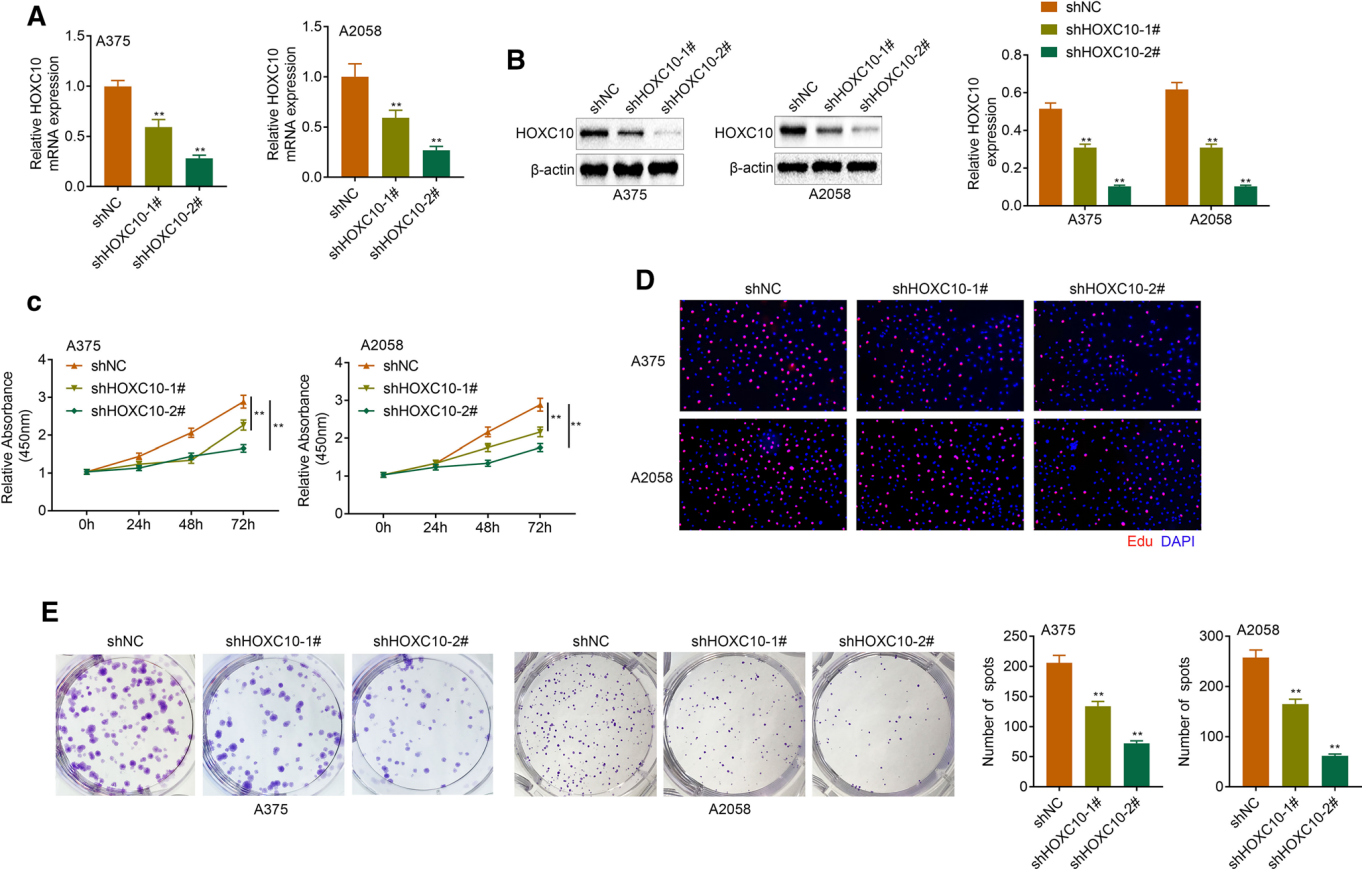
HOXC10 promoted the proliferation ability of A375 and A2058 cells. HOXC10 expression in the A375 and A2058 cells after HOXC10knockdown was estimated through qRT-PCR (**a**) and WB (**b**). Cell proliferation of A375 and A2058 cells after HOXC10knockdown was examined by CCK-8 assay (**c**) and Edu staining (**d**). Clone formation of A375 and A2058 cells after HOXC10knockdown was detected by clone formation assay (**e**). ^**^p < shNC

In order to determine the function role of HOXC10 in melanoma, HOXC10 was silenced in A375 and A2058 cells that express relatively higher expression of HOXC10. HOXC10 mRNA and protein expression was significantly decreased in the A375 and A2058 cells following transfection of shHOXC10-1# or shHOXC10-2#, especially shHOXC10-2# (Fig. 2a, b). The data obtained from CCK-8 and Edu assays revealed that HOXC10 deficiency significantly repressed cell viability and proliferation of A375 and A2058 cells (Fig. 2c, d). Furthermore, HOXC10 silencing suppressed clone formation of A375 and A2058 cells (Fig. 2e). Therefore, these data showed that HOXC10 promoted proliferation of melanoma cells.

### HOXC10 inhibited apoptosis of melanoma cells

**Fig. 3:**
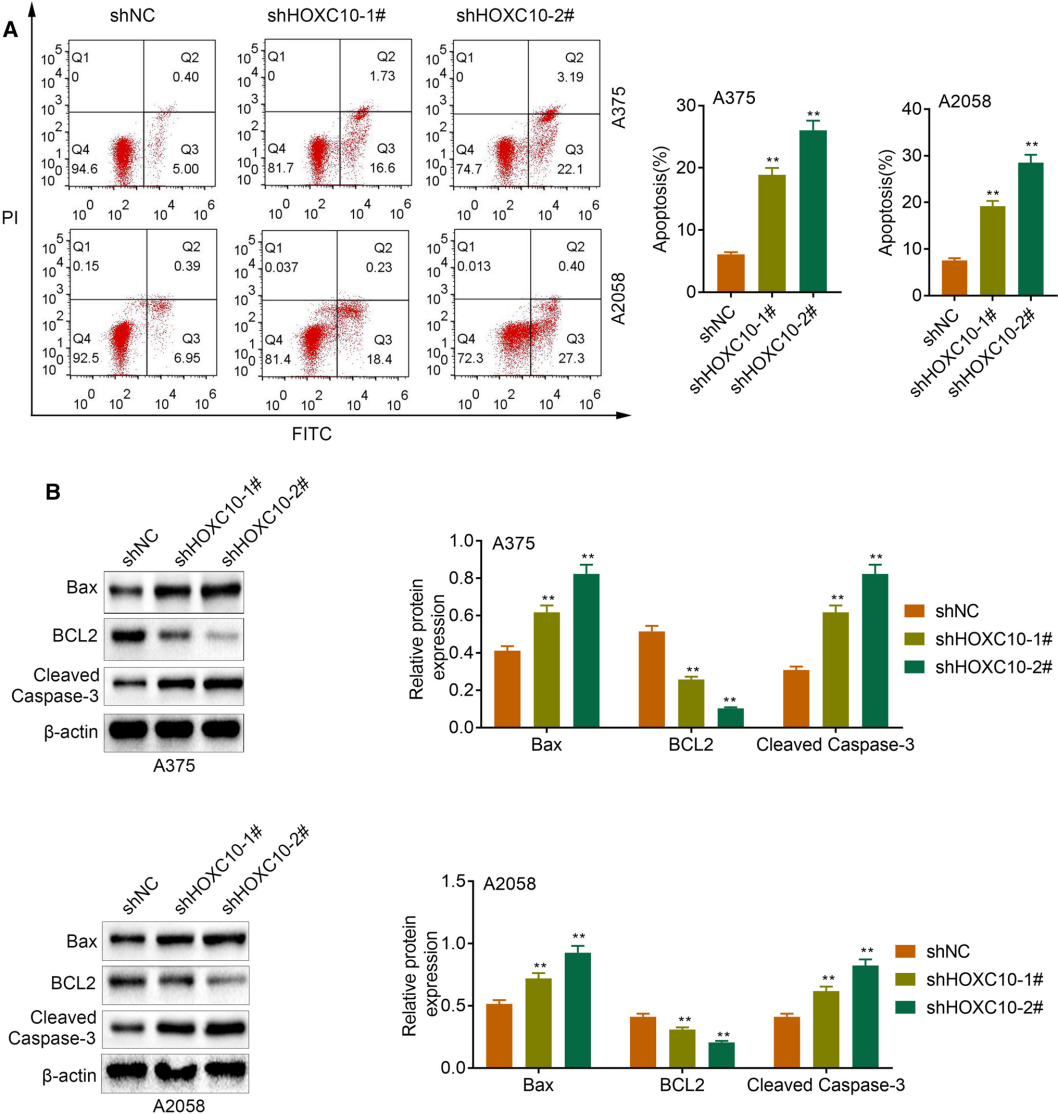
HOXC10 inhibited apoptosis of A375 and A2058 cells. **a** Apoptosis of A375 and A2058 cells after HOXC10knockdown was detected by flow cytometry. **b** The expression of Bax, BCL2 and Cleaved Caspase-3 in the A375 and A2058 cells following HOXC10knockdown was detected examined by WB. ^**^p < shNC

Next, we estimated the impact of HOXC10 on cell apoptosis in melanoma cells. The results of flow cytometry revealed that apoptosis was obviously enhanced in A375 and A2058 cells following transfection of shHOXC10-1# or shHOXC10-2# (Fig. 3a). WB analysis of apoptosis-related proteins showed that HOXC10 silencing enhanced Bax and Cleaved Caspase-3 expression, and reduced BCL2 expression in the A375 and A2058 cells (Fig. 3b). Taken together, these data suggested that HOXC10 inhibited apoptosis of A375 and A2058 cells.

### HOXC10 promoted migration, invasion and EMT of melanoma cells

**Fig. 4:**
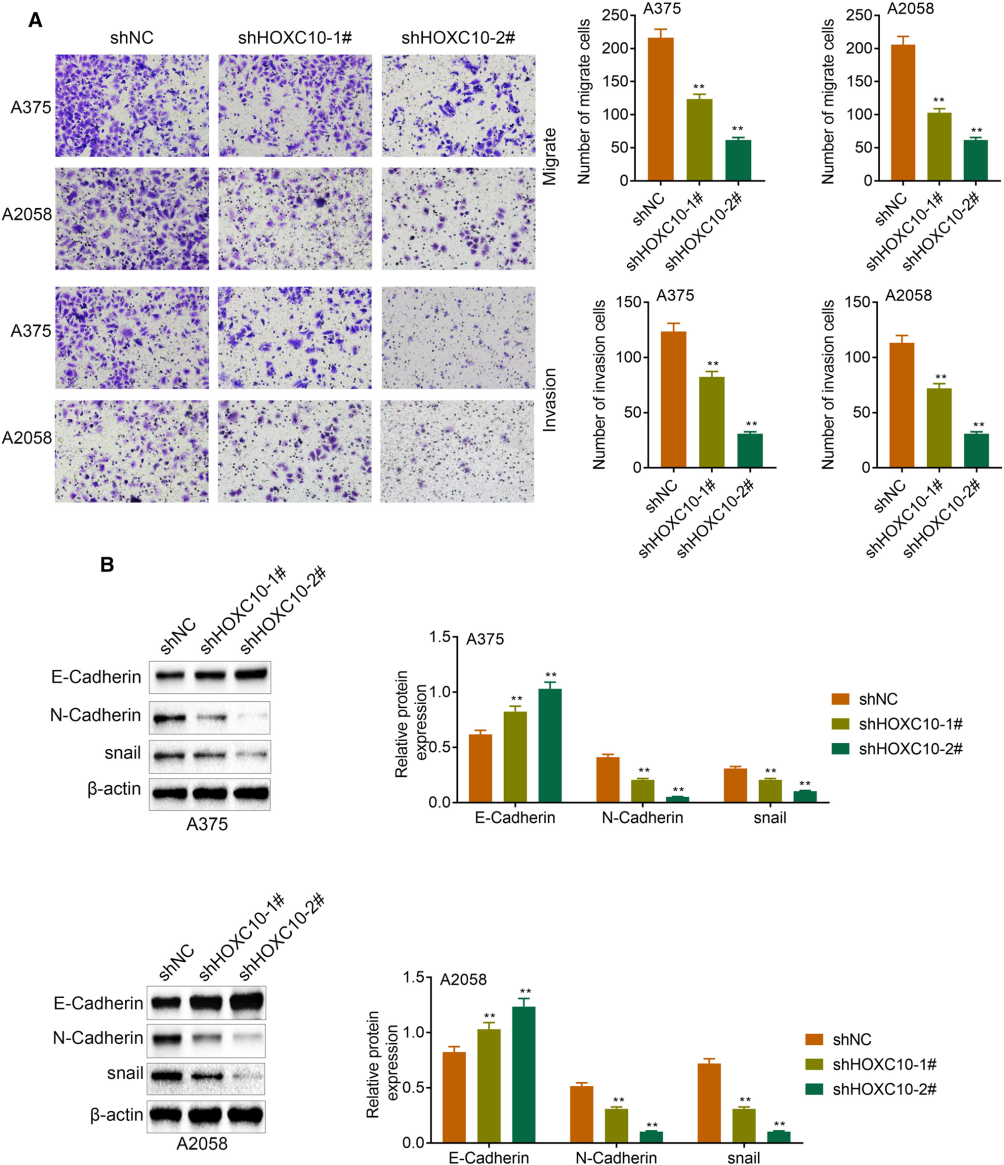
HOXC10 promoted migration, invasion and EMT of A375 and A2058 cells. **a** The ability of migration and invasion of A375 and A2058 cells after HOXC10 knockdown was detected by transwell assay. **b** The expression of E-Cadherin, N-Cadherin and snail in the A375 and A2058 cells following HOXC10 knockdown was detected examined by WB. ^**^p < shNC

We examined migration and invasion of A375 and A2058 cells, showing that HOXC10 deficiency led to a decrease in migration and invasion of A375 and A2058 cells (Fig. 4a). Moreover, the WB analysis of EMT-related proteins showed that HOXC10 deficiency caused an up-regulation of E-Cadherin and a down-regulation of N-Cadherin and snail in the A375 and A2058 cells Fig. 4b. Therefore, these results showed that HOXC10 promoted migration, invasion and EMT of A375 and A2058 cells.

### HOXC10 promoted Slug expression and activated YAP/TAZ signaling pathway

**Fig. 5:**
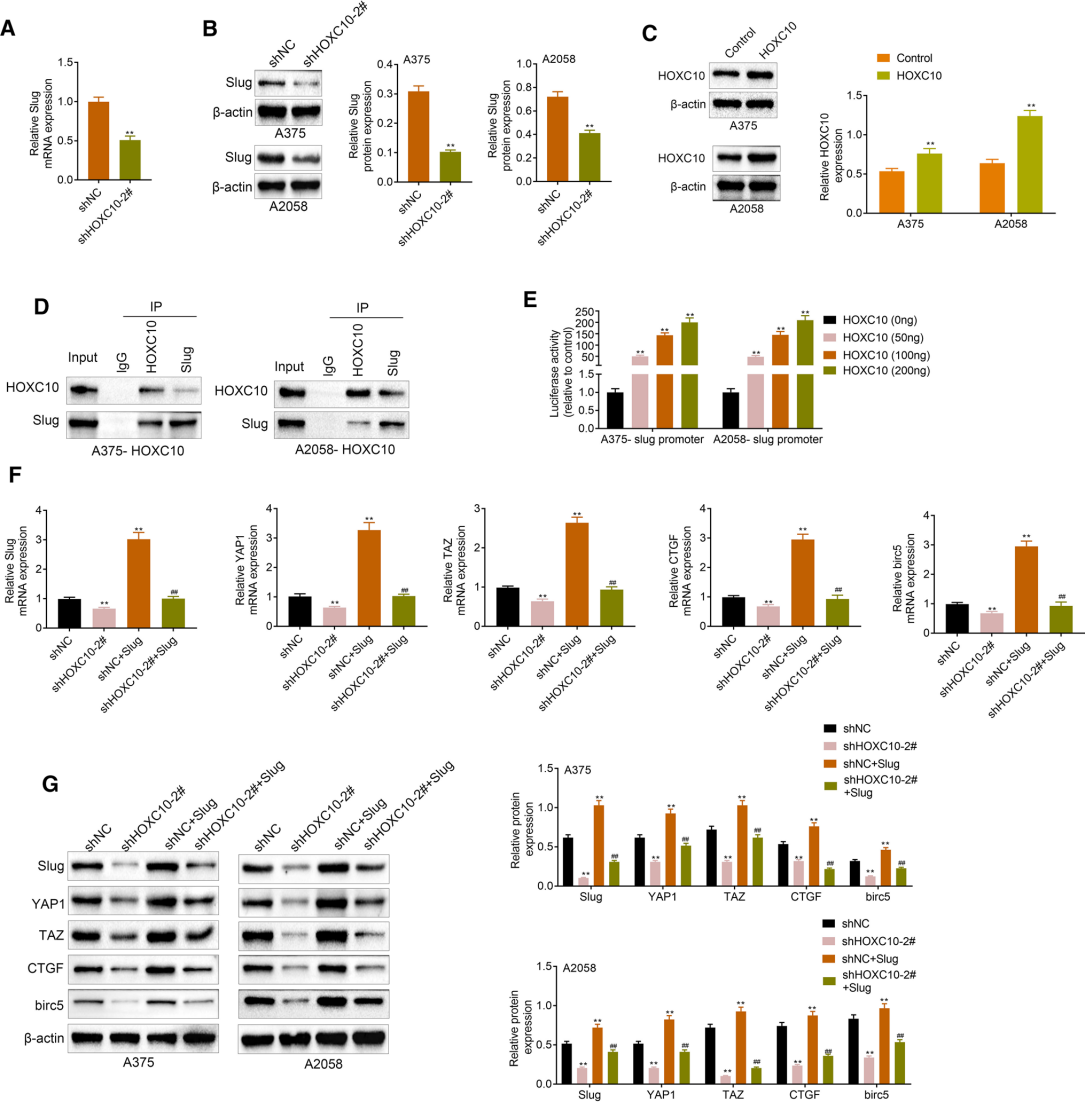
HOXC10 promoted Slug expression and activated YAP/TAZ signaling pathway. The expression of Slug in the A375 and A2058 cells following HOXC10 knockdown was detected examined by qRT-PCR (**a**) and WB (**b**). ^**^p < shNC. The overexpression efficiency of HOXC10 was confirmed (**c**). ^**^p < Control. The relationship between HOXC10 and Slug (promoter) in A375 and A2058 cells was verified by CO-IP (**d**) and luciferase reporter assay (**e**). ^**^p < HOXC10 (0 ng). The expression of Slug, YAP1 and TAZ in the A375 and A2058 cells following HOXC10 knockdown or combined with Slug overexpression was detected examined by RT-qPCR and WB (**f**, **g**). ^**^p < shNC; ^##^*p* < shNC + Slug

We further examined the influence of HOXC10 on the expression of EMT-related protein Slug in A375 and A2058 cells. As shown in Fig. 5a, b, HOXC10 knockdown caused a down-regulation of Slug in the A375 and A2058 cells. Next, the overexpression efficiency of HOXC10 was verified in Fig. 5c. We further determined the relationship between HOXC10 and Slug through CO-IP assay, showing that HOXC10 interacted with Slug in the A375 and A2058 cells (Fig. 5d). Furthermore, HOXC10 activated Slug promoter in A375 and A2058 cells (Fig. 5e). Subsequently, we performed RT-qPCR and WB to estimate the expression of YAP/TAZ signaling pathway-related proteins. In the A375 and A2058 cells, HOXC10 knockdown significantly suppressed the mRNA and protein expression of Slug, YAP1, TAZ, CTGF and birc5. However, overexpression of Slug caused an up-regulation of Slug, YAP1, TAZ CTGF and birc5 in A375 and A2058 cells, which was reversed by HOXC10 knockdown (Fig. 5f, g). Thus, there data confirmed that HOXC10 promoted Slug expression and activated YAP/TAZ signaling pathway.

### HOXC10 knockdown inhibited tumor growth and lung metastasis of melanoma in mice

**Fig. 6:**
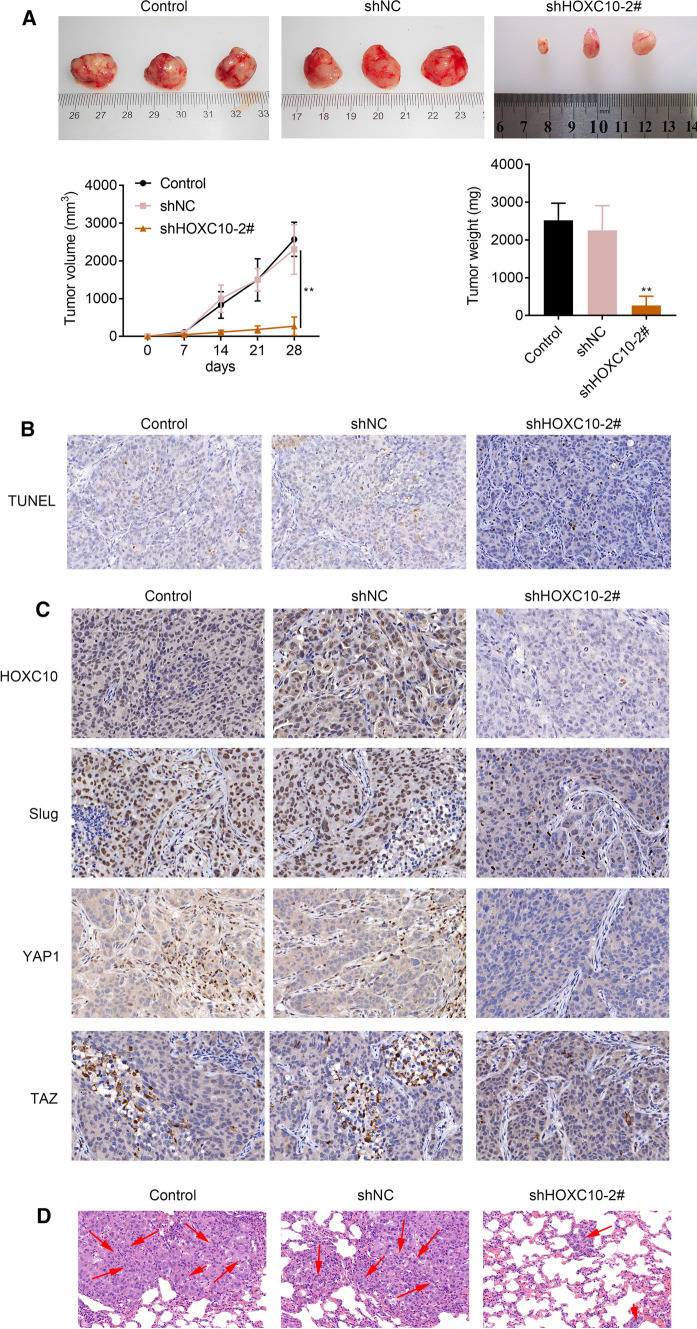
HOXC10 knockdown inhibited tumor growth and lung metastasis of melanoma in mice. **a** The weight and volume of tumor xenografts in Control, shNC and shHOXC10-2# groups were measured. **b** Cell apoptosis in tumor tissues in Control, shNC and shHOXC10-2# groups were assessed by TUNEL staining. **c** The expression of HOXC10, Slug, YAP and TAZ in Control, shNC and shHOXC10-2# groups was estimated through IHC staining. **d** HE staining was performed to estimate the number of metastases in lung tissues in Control, shNC and shHOXC10-2# groups. ^**^P < 0.01 vs. shNC

Finally, to verified the function role of HOXC10 in melanoma, we constructed tumor xenografts of melanoma in mice. Figure 6a showed that HOXC10 knockdown notbaly repressed the weight and volumes of melanoma in mice. We also found that HOXC10 silencing notably enhanced cell apoptosis of tumor tissues in mice (Fig. 6b). Moreover, IHC staining results showed that HOXC10 knockdown caused a down-regulation of HOXC10, Slug, YAP1 and TAZ in the tumor tissues of mice (Fig. 6c). Furthermore, we observed the incidence of lung metastasis in mice, showing that inhibition of HOXC10 attenuated the number of metastasis in lung tissues of mice (Fig. 6d). These deta taken together showed that HOXC10 knockdown inhibited tumor growth and lung metastasis of melanoma in mice.

## Discussion

HOXC10 participated in the development of various cancers. For example, HOXC10 has a oncogenic effect in gastric cancer. HOXC10 is a target of miR-129-5p, and miR-129-5p inhibits gastric cancer development by regulating HOXC10/Cyclin D1 axis [22]. HOXC10 is highly expressed in the poorly differentiated gastric carcinoma cells, and it accelerates proinflammatory cytokines through NF-κB signaling pathway to promote the progression of gastric carcinoma [23]. In liver cancer, HOXC10 expression is markedly decreased. HOXC10 expression is inhibited by miR-221, and inhibition of represses cell proliferation in liver cancer through the activation of MAPK signaling pathway [24]. Upregulation of HOXC10 is closely associated with colorectal cancer progression [25]. However, whether HOXC10 has an oncogenic effect in melanoma is currently unclear. Here, we first determined the biological role of HOXC10 in melanoma. HOXC10 was highly expressed in the melanoma tissues and cells. These data showed that HOXC10 may has a crucial role in the progression of melanoma.

Our in vitro assays have found that HOXC10 silencing inhibited proliferation and clone formation, and enhanced apoptosis of melanoma cells. Additionally, HOXC10 deficiency decreased melanoma cell migration, invasion and EMT. Taken together, these findings indicated that HOXC10 promoted melanoma development.

Slug participates in the progression of EMT that occurs during melanocyte emigration from the neural crest. Slug regulates invasion and metastasis in cutaneous melanoma through the cooperation of TSP-1 with FGF-2 and VEGF/VEGFR-1 [26]. Slug knockdown attenuates invasive behavior and blocks SPARC-medicated promotion of cell migration in melanoma [27]. Slug has a crucial role in the regulation of cellular network that involved in the response to DNA damage, and knockdown of Slug may contribute to enhance the radiation sensitivity of melanoma cells [28]. In this work, we found that HOXC10 overexpression enhanced Slug expression, while HOXC10 silencing reduced Slug expression in melanoma cells. HOXC10 interacted with Slug, and activated Slug promoter. Thus, these data suggested that HOXC10 promoted the progression of melanoma by activating Slug.

YAP/TAZ drives cancer cell survival, proliferation, invasive, migration and metastasis [29]. The study of Feng et al. has found that the protein expression of YAP and TAZ are positively correlated with malignant melanoma, and high expression of YAP/TAZ is correlated with adverse outcomes in the postoperative melanoma patients [30]. Inhibition of TPC2 activates genes regulated by YAP/TAZ signaling pathway, and thus enhances the aggressiveness of melanoma [31]. Consistently, we also found the involvement of YAP/TAZ signaling pathway in melanoma. Slug overexpression activated YAP/TAZ signaling pathway in melanoma cells. HOXC10 knockdown reversed the inhibiting effect of Slug up-regulation on YAP/TAZ signaling pathway. Finally, our in vivo assays demonstrated that inhibition of HOXC10 significantly repressed tumor growth and lung metastasis of melanoma in the tumor xenografts mice. Additionally, HOXC10 deficiency repressed tumor growth and lung metastasis of melanoma through the inhibition of Slug and YAP/TAZ signaling pathway.

In conclusion, this work demonstrated that HOXC10 promoted growth and migration of melanoma by regulating Slug to activate the YAP/TAZ signaling pathway. Therefore, this study suggests that inhibition of HOXC10 has therapeutic potential in melanoma.

## Data Availability

All data generated or analyzed during this study are included in this published article.
